# The Political Environment of Global Oral Health: Now is the Moment to Improve Equity

**DOI:** 10.5334/aogh.4284

**Published:** 2023-10-09

**Authors:** Jane Barrow, Ashiana Jivraj, Gene Bukhman, Mickey Chopra, Marko Vujicic, Habib Benzian

**Affiliations:** 1 Harvard School of Dental Medicine, Boston, US; 2 Commonwealth Care Alliance, Boston, MA, US; 3 Center for Integration Science, Brigham & Women’s, US; 4 Harvard Medical School, Boston, US; 5 Global Solutions Lead for Service Delivery in the Health Nutrition and Population Global Practice, World Bank, Washington, DC, US; 6 Health Policy Institute, American Dental Association, Chicago, US; 7 WHO Collaborating Center Quality Improvement & Evidence Based Dentistry, NYU College of Dentistry, New York, US; 8 Stellenbosch Institute of Advanced Study (STIAS), Stellenbosch, South Africa

**Keywords:** global oral health, medical-dental integration, holistic health, global equity, universal health coverage, political economy

## Abstract

Harvard School of Dental Medicine’ Initiative to Integrate Oral Health and Medicine, in collaboration with Harvard’s Center for Integration Science and Global Health Institute, convened global experts in integration science and medical dental integration specifically, to call attention to the need for universal health coverage concepts that incorporate essential oral health services and thereby address equity and population health gaps.

Across the globe, there are many innovative financial, clinical and educational programs that strive to provide comprehensive and universal healthcare that includes oral health. The goal of the symposium was to showcase successful examples of programs and policies that are improving health and quality of life, particularly for communities suffering disparities, through the integration of oral healthcare into primary and secondary levels of care.

Symposium participants ranged from ministers of health to village healthcare workers to academics, who shared their successes and challenges integrating medical and oral healthcare. However, despite innovative examples spanning integration of care for infectious and noncommunicable diseases as well as social determinants, more work is required to: heighten awareness of the essentialism of oral health; strengthen the evidence for effective oral healthcare; and highlight the opportunity to improve health and equity through interprofessional collaboration.

This commentary presents the key points from a subject matter expert discussion, theorizing through the lens of political economy about the challenges to advance the integration of oral healthcare within universal healthcare, and how the inspiring examples of success showcased throughout during the symposium surmounted systemic and cultural barriers to holistic care.

## Introduction

Harvard School of Dental Medicine, in collaboration with the Harvard Center for Integration Science and the Harvard Global Health Institute, convened global experts in integration science—and medical-dental integration specifically—to discuss how oral health can be incorporated into universal health coverage policies to help reduce population health gaps. The goal of the symposium was to showcase successful examples of policies and programs that improve health and quality of life through the integration of oral healthcare into all levels of care. Despite many innovative examples, large-scale progress in expanding coverage for essential oral healthcare is lagging. This commentary reviews key aspects of a panel discussion on “The Political Economy and Integrating Medical and Oral Healthcare” (see recording link in reference list). It outlines challenges to advance the integration of oral healthcare identified by the panel and highlights promising practices showcased through the symposium.

## A Snapshot of Inequalities in Oral Healthcare

The new WHO Global Strategy for Oral Health clearly recognizes that “achieving the highest attainable standard of oral health is a fundamental right of every human being [1,2].” However, the current momentum for Universal Health Coverage (UHC) often does not integrate oral healthcare. No other disease group affects such a vast number of people. An estimated 3.5 billion individuals (46% of the global population) live with untreated oral diseases, furthered by limited access to prevention, care, and rehabilitation [3]. Approximately 80% of global direct dental expenditure benefits only around 20% of the world’s population [3]. High-income countries allocate a staggering 800 times more funds to oral healthcare compared to low-income countries [3]. There are 20 times more oral health professionals in high-income nations as compared to low-income [3]. The burden of disease patterns, however, are similar across nations, with the highest prevalence shouldered by poor, marginalized, and disadvantaged populations [3]. To shift the curve, especially to meet the Sustainable Development Goal of 80% of the world’s population having access to essential healthcare by 2030, there needs to be significant, cross-sectoral, and accelerated action of governments, professional organizations, funding agencies, communities, and many others [4].

## Barriers to Advancing Universal Access to Oral Healthcare

Although current coverage gaps and high out-of-pocket payments tell a compelling story about the need for reform, visibility and public acknowledgement for this need is comparatively low. Most non-communicable diseases (NCDs) remain under the shadow of the dominant NCD framing that focuses on five main diseases. The current oral health crisis and its impact on overall wellbeing needs to be illuminated. Today’s healthcare marketplace driven by profit-making and economic incentives results in exorbitant costs that lead to exclusion of populations who are not able to pay; and this is a major barrier to expanding access and coverage for oral healthcare. The promise of UHC requires significant financial investments of governments and partners to address all aspects of health. Rather than reshuffling budgets from one issue to another, new investment needs to be mobilized with clear aims to result in lower individual expenditures and greater cost-effectiveness. The oral and public health communities need to partner to understand both the barriers and drivers of change to design health systems that deliver on the promise of health and oral health for all [5].

Solution design starts with appropriate policy interventions. Today, there is a deep inculcated separation of care delivery and financing. The gap has led to an internalized idea of neglect over decades [6]. Alignment of care and financials will require a deliberate strategy that brings together individuals in power to develop and agree on realistic solutions. The current state of oral health globally has been further worsened by a lack of consensus on high-impact and cost-effective solutions at a population, community, and individual level, though the WHO and others are currently working on defining “Best Buy” interventions to address oral diseases. In addition, oral health solutions need to be presented to decision-makers in terms of return on investment. Creating inclusive oral health financing policies also requires rethinking how to frame arguments and advocacy. For example, in the US, 30% of low-income adults say that oral disease limits employment opportunities [7]. Framing oral health within a national economy context will garner new allies, beyond the current sources of funding [8].

Scarcity of oral health professionals is also a challenge. Community engagement leveraging existing non-dental workforces may broaden delivery capabilities and diminish costs. Harnessing alternative workforce models can promote task shifting and expand oral health service delivery capacity. Models that shift away from focusing mainly on dentists may be effective in managing high costs affiliated with traditional care delivery models. Tailoring the care to the needs of the population is essential to optimize investments and allows for a high-level of integrated care.

Emphasis on upstream policy measures that reduce risks common to NCDs and oral health will be equally crucial. The taxes on sugar-sweetened beverages in Mexico and other countries show that such measures are possible, despite the power of industries and political interest groups. The oral health community so far doesn’t drive such policy reforms but could lend a potentially impactful and supportive voice.

Integrated solutions and policy approaches require involving economists, health services researchers, social scientists, and other discipline experts. Understanding and integrating implementation and political processes can shape better service models that are contextually relevant and impactful. Each community and nation may require different models to address their specific challenges and that fit within their respective context. The energy that exists around the WHO Global Oral Health Action Plan offers a unique and unprecedented opportunity to advance toward universal health coverage.

## Summary

This special collection in *Annals of Global Health*, therefore, touches on the momentum and energy that exists globally around reforming and integrating healthcare services to improve health. The experts attending this symposium brought perspectives that evolved over many years of service in policy, clinical care, research, academia, program development and implementation, and interprofessional collaboration in behavioral health, medicine, and dentistry. This issue looks to showcase the many innovative financial, clinical, and educational programs that strive to provide comprehensive and universal healthcare, including oral, behavioral and primary healthcare.
